# Combination prevention and HIV: a cross-sectional community survey of gay and bisexual men in London, October to December 2016

**DOI:** 10.2807/1560-7917.ES.2019.24.25.1800312

**Published:** 2019-06-20

**Authors:** Louise Logan, Ibidun Fakoya, Alison Howarth, Gary Murphy, Anne M Johnson, Alison J Rodger, Fiona Burns, Anthony Nardone

**Affiliations:** 1 HIV and STIs Department, Public Health England, London, United Kingdom; 2 Institute for Global Health, University College London, United Kingdom; 3 Laboratory Services, National Infections Service, Public Health England, London, United Kingdom; 4 Royal Free London NHS Foundation Trust, London, United Kingdom

**Keywords:** HIV, PrEP, chemsex, men who have sex with men - MSM, MSM, convenience sample, oral fluid testing, United Kingdom, UK, sexually transmitted infections, HIV infection, epidemiology

## Abstract

**Background:**

Men who have sex with men (MSM) are at risk of HIV and are an important population to monitor and ameliorate combination prevention efforts.

**Aim:**

To estimate HIV prevalence and identify factors associated with frequent HIV testing (≥ 2 HIV tests in the last year) and pre-exposure prophylaxis (PrEP) use among MSM in London.

**Methods:**

For this cross-sectional study, MSM recruited from 22 social venues provided oral-fluid samples for anonymous HIV antibody (Ab) testing and completed a questionnaire. Factors associated with frequent HIV testing and PrEP use were identified through logistic regression.

**Results:**

Of 767 men recruited, 545 provided an eligible oral specimen. Among these, 38 MSM (7.0%) were anti-HIV positive including five (13.2%; 5/38) who reported their status as negative. Condomless anal sex within the previous 3 months was reported by 60.1% (412/685) men. Frequent HIV testing was associated with, in the past year, a reported sexually transmitted infection (adjusted odds ratio (AOR): 5.05; 95% confidence interval (CI): 2.66–9.58) or ≥ 2 casual condomless partners (AOR 2–4 partners: 3.65 (95% CI: 1.87–7.10); AOR 5–10 partners: 3.34****(95% CI: 1.32–8.49). Age ≥ 35 years was related to less frequent HIV testing (AOR 35–44 years: 0.34 (95% CI: 0.16–0.72); AOR ≥ 45 years: 0.29 (95% CI: 0.12–0.69). PrEP use in the past year was reported by 6.2% (46/744) of MSM and associated with ≥ 2 casual condomless sex partners (AOR: 2.86; 95% CI: 1.17–6.98) or chemsex (AOR: 2.31; 95% CI: 1.09–4.91).

**Conclusion:**

This bio-behavioural study of MSM found high rates of behaviours associated with increased risk of HIV transmission. Combination prevention, including frequent HIV testing and use of PrEP, remains crucial in London.

## Introduction

Combination prevention is an effective approach to address the HIV epidemic, but requires implementation of a range of interventions including condom use, high levels of HIV testing, early initiation of antiretroviral therapy (ART) and, more recently, appropriate pre-exposure prophylaxis (PrEP) use in populations at higher risk of HIV [1-3].

In the United Kingdom (UK), men who have sex with men (MSM) remain the population at highest risk of acquiring HIV. In 2016, there were 2,236 new HIV diagnoses among MSM in England, of those nearly half (49%; 1,096) lived in London [4]. Of all MSM living with HIV in the UK, one in 10 were estimated to be unaware of their HIV infection status in 2016 compared with one five in 2010 [5,6]. Significant declines in the number of new diagnoses in MSM attending sexual health (SH) clinics in London have been observed between 2014 and 2015 and attributed to increased repeat HIV testing alongside decreased time between HIV diagnosis and initiation of ART as well as increased use of PrEP [7,8]. More frequent testing in MSM is also likely to result in earlier diagnosis, decreasing the time from infection to suppressive treatment and thereby preventing onward transmission.

Rates of HIV testing in the MSM population in the UK are high and increasing. In London, the Gay Men’s Sexual Health survey (GMSHS) allowed us to observe that the proportion of men reporting having had an HIV test in the last year increased from 26% in 2000 to 60% in 2013 [9]. National guidelines recommend that MSM should test annually and those having condomless anal sex with new or casual partners should have an HIV test every 3 months [6,10]. Rates of condomless sex have increased, with 43% of MSM participating in the GMSHS reporting condomless anal intercourse in the previous 12 months in 2000 compared with 53% in 2013 [9].

PrEP is highly effective in protecting individuals from HIV acquisition, [11,12]; however, at the time of this study it was not provided by the National Health Service (NHS) in England and could only be sourced via private prescriptions or bought online. For those sourcing PrEP privately, the recommended clinical monitoring before initiating and while on PrEP was provided by some NHS SH services. Since the time of this study PrEP has become available free of charge to 26,000 eligible people through the PrEP IMPACT trial, enrolment for which began through SH clinics in England in October 2017 [13].

There is a lack of current data on the rate of PrEP use among MSM in London alongside other measures of combination prevention including condom use and HIV testing which are crucial to understand the impact and implementation of combination prevention in MSM in this city. We report the latest iteration in 2016 of the London GMSHS, a community-based questionnaire survey of MSM conducted periodically since 1997 (last conducted in 2013) which has included the collection of anonymous oral fluid specimens to allow testing for HIV since 2000 [9]. The aim of this survey was to better understand the prevention needs of MSM in London by estimating the diagnosed and undiagnosed HIV prevalence and identify factors associated with key prevention interventions, such as HIV testing and PrEP use.

## Methods

### Study population and data collection

Methods used in this cross-sectional study have been described previously [9,14] but were adapted for this 2016 survey. A total of 22 pubs, bars, clubs and saunas in London and Greater London were included in the survey, primarily selected from a list of those used in previous GMSHS surveys. Internet searches were used to identify newer venues and replace those that had closed. Of the 59 venues identified, 15 venues, including all sex on premises venues and those in areas with few venues (North and West London), were approached and from the remaining 44 venues, 20 were randomly selected and approached. Where possible, venues that declined to participate were replaced with a similar alternative as geographically close to the original as possible.

The paper questionnaire (Supplement S1) was updated to include questions on PrEP use and the recognition and influence of the London HIV prevention programme (LHPP) campaigns which aimed to increase HIV testing, condom use and adoption of safer sexual behaviours in MSM and people of black African ethnicity.

Participants were recruited between 16 October and 9 December 2016 by trained fieldworkers who visited venues multiple times over a range of pre-arranged dates and times. The purpose of the survey, oral fluid collection and inclusion criteria were explained to potential participants, information sheets were provided and verbal consent was obtained. All data were collected anonymously and information on how to access named testing was included in the information sheet. Men who were aged 18 years and over, identified as gay or bisexual or who had had sex with a man within the last year were eligible to participate. Individuals completed the questionnaire themselves and placed it into the tamper-proof envelope provided.

### Oral fluid collection and device validation

All participants were invited to provide an oral fluid specimen including those who knew their HIV status to be positive. Fieldworkers were trained to explain that even when positive status was already known, obtaining samples would enable estimation of overall prevalence as well as undiagnosed HIV prevalence. Oral fluid specimens were collected using the Intercept i2heTM (Orasure Technologies) device which replaced the OraSure kitTM devices used in previous surveys as these were no longer available in the European Union. The unmarked device was placed under the tongue for between 2 and 15 min until the indicator changed colour. Completed specimen tubes were sealed and placed into tamper-proof envelopes with the completed questionnaire. All specimens were transported to the National Infections Service, London within 7 days of collection to ensure they were processed within the 21-day ambient storage window. Specimens were tested for HIV antibody using the in-house IgG antibody-capture enzyme-linked immunosorbent assay (GACELISA) assay. Reactive specimens (optical density/cut off ratio (OD/CO) > 1.0) were presumed reactive, repeat tested and confirmed by western blot. All specimens were tested for total IgG concentration to ensure specimen quality and specimens with < 0.2 mg/L IgG were excluded from the results. All GACELISA HIV antibody negative specimens from participants reporting their HIV status as positive were submitted for western blot testing to confirm the GACELISA result.

The new oral fluid collection device was validated to ensure comparable performance of the GACELISA assay with previous surveys that used the Orasure kitTM collection device. A total of 119 specimens (102 from known HIV positive patients and 17 from known negative PHE staff volunteers) were collected and tested for total IgG concentration and HIV antibodies. Of the negative specimens all had CO/OD of < 1.0, of the positive specimens one was excluded due to low IgG (concentration < 0.2 mg/L) and of the rest 92.1% (93/101) had CO/OD > 1.0. These results were similar to devices employed in previous GMSHS and thus sufficient to proceed with the Intercept i2heTM for the 2016 surveys.

### Ethical approval 

Ethical approval for the study was granted by the London Harrow Research Ethics Committee (REC reference 00/0158). 

### Statistical analysis

Data were double entered into a Microsoft Access 2010 database and validated and analysed in Stata v13.1. Binary logistic regression was used to analyse associations between demographic and behaviour variables and the primary outcomes. Crude odds ratios (OR) were calculated and associations with a p value < 0.10 were included in the multivariate regression models. Associations are reported as OR or adjusted OR (AOR) with 95% confidence intervals (CI) and p values. Participants reporting their status as HIV positive and not testing in the past year were excluded from analyses on HIV testing. Collected variables were combined to create the derived variables of chemsex (participants who answered yes to using ketamine, mephadrone, gammahydroxybutrate or methamphetamine before or during sex) and frequent testers (participants that recorded two or more HIV tests in the previous 12 months).

## Results

Questionnaires were collected from 767 men of whom 23 were excluded either because they did not self-identify as MSM (n = 18) or had already participated in the survey (n = 5); the remaining 744 men were included in the following analyses. The median age of participants was 33 years (interquartile range (IQR): 20–65) and the majority were of white ethnicity (79.6%), UK born (57.3%), in current employment (89.1%) and had 2 or more years of education after the age of 16 years (73.9%). A total of 412 (60.1%) men reported condomless anal sex within the previous 3 months and a similar proportion (59.3%; i.e. 301/508 with information available) reported at least one casual condomless anal sex partner in the previous 12 months. In the previous 12 months, a total of 46 (6.2%) reported using PrEP, 147 (19.8%) reported having being diagnosed with a sexually transmitted infection (STI) and 154 (20.7%) reported engaging in chemsex (Table 1). There were no significant differences between men recruited in different venue types but saunas had the highest proportion of men over 50 years old (22.2%; 10/45) compared with clubs and bars/pubs where 5.3% (9/169) and 8.5% (44/520) were aged over 50 years respectively.

**Table 1:** Demographic characteristics study participants, London, United Kingdom, 16 October–9 December 2016 (n = 744)

Demographic characteristic (total number of respondents with information available)	n	%
Age group in years (N = 737)		
18–24	78	10.6
25–29	169	23.0
30–34	162	22.0
35–39	130	17.6
40–44	84	11.4
45–49	50	6.8
≥ 50	64	8.7
Ethnicity (N = 740)		
White	589	79.6
Black	25	3.4
South East Asian	16	2.2
South Asian	26	3.5
Latin American	28	3.8
Mixed	39	5.3
Other	17	2.3
World region of birth (N = 737)		
United Kingdom	422	57.3
Rest of Europe	164	22.3
Americas and the Caribbean	66	9.0
Sub-Saharan Africa	14	1.9
South East and East Asia	16	2.2
Other	55	7.5
Currently employed (N = 742)		
No	81	10.9
Yes	661	89.1
Education after 16 years of age (N = 740)		
None	49	6.6
< 2 years	109	14.7
≥ 2 years	547	73.9
Still in full time education	35	4.7
Last condomless sex (N = 685)		
Last 3 months	412	60.1
3–12 months ago	113	16.5
> 12 months ago	160	23.4
Number of casual^a^ condomless partners in the last 12 months (N = 508)		
0	207	40.8
1	155	30.5
2–4	73	14.4
5–10	41	8.1
≥ 11	32	6.3
Had an STI in the last 12 months (N = 743)		
No	596	80.2
Yes	147	19.8
Chemsex^b^ in the last 12 months (N = 744)		
No	590	79.3
Yes	154	20.7
Had used PrEP in the last 12 months (N = 744)		
No	698	93.8
Yes	46	6.2
Venue of recruitment (N = 741)		
Bar/pub	522	70.5
Club	172	23.2
Sauna	47	6.3

PrEP: pre-exposure prophylaxis; STI: sexually transmitted infection.

^a^ Casual partner is defined as a partner with whom the respondent has had sex with only once.

^b^ Chemsex is defined as use of any of the following drugs before or during sex: gammahydroxybutrate (GHB) ketamine, mephadrone, or methamphetamine in the previous 12 months.

### Oral fluid results and undiagnosed HIV

Of the 744 men included in this analysis, 585 (78.5%) provided an oral fluid specimen. Of these, six (1.03%) had total IgG concentrations too low for testing and a further 34 (5.8%) were excluded due to a laboratory processing error leaving 545 for analysis. A total of 38 (7.0%) specimens were positive for HIV 1 antibodies. Self-reported HIV status and oral fluid results were compared and of the 474 men who reported their HIV status as negative, five (1.0%) were HIV antibody positive indicating undiagnosed HIV infections. Of all those with HIV antibody positive oral fluid specimens in this study population (n = 38), five men (13.2%; 95% CI: 2.4–24.0%) were unaware of their infection and had reported their status as negative. Of the 35 men who self-reported as HIV positive, four (11.5%) were HIV antibody negative on both the GACELISA and western blot assays likely indicating reduced oral fluid test sensitivity in the setting of longstanding ART [15]. 

Men who reported their status as HIV positive were significantly less likely to provide a specimen than men reporting their status as negative with rates of 61.4% (35/57) and 78.5% (474/604) respectively (p = 0.005).

### HIV testing

Rates of HIV testing were high with 96.2% (710/738) of men reporting ever having an HIV test and 69.7% (514/738) having had a test in the previous 12 months. The number of HIV tests undertaken in the previous year was reported by 700 men; In order to eliminate from the analysis those who had not had an HIV test because they know they are positive, men who reported their status to be positive and had not had a test in the last year were excluded, leaving a total of 668 men. Of these, 86 (12.9%) reported having four or more tests. Of the 125 HIV negative men who reported two or more casual condomless partners in the previous 12 months, 57 (45.6%) reported four or more HIV tests in the same time period. The majority of men, 77.0% (565/734) reported attending a SH clinic for their last HIV test.

The proportion of men who reported having two or more HIV tests in the last 12 months was 48.7% (325/668). Multivariate logistic regression analysis comparing men reporting two or more tests with those reporting fewer than two in the previous 12 months, revealed that men aged 35 years and over were significantly less likely to have tested more than once compared with younger men (AORs: 0.34 and 0.29 for MSM aged 35–44 years and ≥45 years respectively). Men diagnosed with an STI in the previous 12 months were significantly more likely to report frequent HIV testing (AOR: 5.05) than those not reporting an STI. Men reporting between more than two casual condomless partners in the previous 12 months (AORs: 3.65, 3.34 and 9.47 for MSM having 2–4, 5–10 and >10 such partners respectively) were also more likely to report frequent testing compared with men reporting one or no casual condomless partner in the previous 12 months. Frequency of testing was not associated with ethnicity, world region of birth, employment status, years of education, chemsex participation or PrEP use (Table 2).

**Table 2:** Logistic regression analyses to assess the association of certain characteristics with reporting two or more HIV tests in the last 12 months^a,b^, London, United Kingdom, 16 October–9 December 2016

Characteristic (total number of respondents with information available)	n	%	Univariate analysis		Multivariate analysisi	
			OR (95% CI)	p value	AOR (95% CI)	p value
Age category in years (N = 663)						
18–24 (n = 69)	41	59.4	Reference	<0.001	Reference	0.003
25–34 (n = 306)	170	55.5	0.85 (0.50–1.45)		0.61 (0.30–1.23)	
35–44 (n =191)	78	40.8	0.47 (0.27–0.83)		**0.34 (0.16–0.72)**	
≥ 45 (n = 97)	33	34.0	0.35 (0.19–0.67)		**0.29 (0.12–0.69)**	
Ethnicity (N = 664)						
White (n = 523)	247	47.2	Reference	0.255	NA	NA
Black (n = 24)	13	54.2	1.32 (0.58–3.00)		NA	
South East & East Asian (n = 39)	24	61.5	1.79 (0.92–3.49)		NA	
Latin American (n = 26)	13	50.0	1.12 (0.51–2.46)		NA	
Other (n = 52)	27	51.9	1.21 (0.68–2.13)		NA	
World region of birth (N = 662)						
United Kingdom (n = 379)	177	46.7	Reference	0.184	NA	NA
Europe (n = 146)	72	49.3	1.11 (0.76–1.63)		NA	
Americas & Caribbean (n = 61)	34	55.7	1.44 (0.83–2.48)		NA	
Sub-Saharan Africa (n = 12)	10	83.3	5.71 (1.23–26.39)		NA	
South East & East Asia (n = 26)	15	57.7	1.56 (0.70–3.48)		NA	
Other (n = 38)	17	44.7	0.92 (0.47–1.81)		NA	
Employment status (N = 666)						
Employed (n = 603)	302	50.0	1.74 (1.02–2.99)	0.006	1.28 (0.61–2.66)	0.137
Years of education after 16 years of age (N = 664)						
None (n = 36)	18	50.0	Reference	0.973	NA	NA
< 2 years (n = 98)	48	49.0	0.960 (0.447–2.061)		NA	
≥ 2 years (n = 498)	244	49.0	0.961 (0.488–1.890)		NA	
Still in full time education (n = 32)	14	43.8	0.778 (0.299–2.024)		NA	
Had an STI in the last year (N = 667)						
Yes (n = 122)	99	81.0	6.08 (3.74–9.86)	<0.001	**5.05 **(**2.66**–**9.58**)	<0.001
Number of casual^c^ condomless partners in last year (N = 456)						
0 (n = 194)	75	38.7	Reference	<0.001	Reference	0.010
1 (n = 144)	67	46.5	1.38 (0.89–2.14)		1.26 (0.79–2.02)	
2–4 (n = 65)	48	73.8	4.48 (2.40–8.36)		**3.65 (1.87**–**7.10)**	
5–10 (n = 33)	25	75.8	4.96 (2.13–11.57)		**3.34 (1.32**–**8.49)**	
> 10 (n = 20)	18	90.0	14.28 (3.22–63.31)		**9.47 (1.92**–**46.62)**	
Chemsex^d^ (N = 668)						
Yes (n = 132)	81	61.0	1.90 (1.289–2.81)	0.005	1.23 (0.70–2.14)	0.572
PrEP use (N = 660)						
Yes (n = 37)	30	81.0	4.89 (2.12–11.30)	<0.001	1.75 (0.64–4.77)	0.046

AOR: adjusted odds ratio; CI: confidence interval; NA: not applicable; OR: odds ratio; PrEP: pre-exposure prophylaxis; STI: sexually transmitted infection.

^a^ 32 men who self-reported as HIV positive and had not had a test within the last year were excluded from this analysis.

^b^ Adjusted model contains a total of 447 observations.

^c^ Casual partner is defined as a partner with whom the respondent has had sex with only once.

^d^ Chemsex is defined as use of any of the following drugs before or during sex; mephadrone, ketamine, gammahydroxybutrate (GHB) or methamphetamine in the last 12 months.

### Pre-exposure prophylaxis use

Overall, 46 of the 744 MSM (6.2%) reported using PrEP in the previous 12 months of whom 24 were using PrEP at the time of the survey. A total of 13 men did not report their source of PrEP, but of those that did (n = 33), the most frequently reported source was the Internet with 14 buying it online. Concerning the past 12 months, six PrEP users reported not having attended a SH clinic and five reported not having an HIV test. Of the 492 men for whom we had a negative oral fluid result and data on the last condomless sex and PrEP use, 463 reported not using PrEP. Of these, 274 (59.2%) reported condomless sex in the previous 3 months.

In the multivariate analysis men reporting two or more casual condomless partners (AOR: 2.86) or chemsex in the last year (AOR: 2.31) were significantly more likely to have used PrEP. In the univariate analysis, those who reported having one or more STI diagnoses in the previous 12 months were more likely to report using PrEP (21/143; 14.7%) than those who had not been diagnosed with an STI (25/588; 4.3%) (OR: 3.88); but the association did not remain in the multivariate model (AOR: 1.58; 95% CI: 0.72–3.45). Men reporting two or more HIV tests in the last year was also significantly associated with PrEP use in the univariate analysis, but there was no evidence of association in the multivariate analysis (AOR: 2.13; 95% CI: 0.93-4.88). There were no significant differences in PrEP use by age, ethnicity, education level, employment status or serosorting behaviour (Table 3).

**Table 3:** Logistic regression analyses to assess the association of certain characteristics with self-reported pre-exposure prophylaxis use in the previous 12 months^a^, London, United Kingdom, 16 October–9 December 2016

Characteristic (total number of respondents with information available)	n	%	Univariable analysis		Multivariable analysis	
			OR (95% CI)	p value	AOR (95% CI)	p value
Age category in years (N = 725)						
< 25 (n = 77)	5	6.5	Reference	0.885	NA	NA
25–39 (n = 452)	30	6.6	1.02 (0.38–2.73)		NA	
≥ 40 (n = 196)	11	5.6	0.86 (0.29–2.55)		NA	
Ethnicity (N = 729)						
White (n = 582)	33	5.7	Reference	0.161	NA	NA
Non-white (n = 147)	13	8.8	1.61 (0.83–3.15)		NA	
Year of education after 16 (N = 728)						
None (n = 44)	4	9.1	Reference	0.631	NA	NA
< 2 years (n = 105)	4	3.8	0.40 (0.09–1.66)		NA	NA
≥ 2 years (n = 545)	36	6.6	0.71 (0.24–2.09)		NA	NA
Still in education (n = 34)	2	5.9	0.63 (0.11–3.63)		NA	NA
Employment status (N = 730)						
Employed (n = 652)	40	6.1	0.78 (0.32–1.91)	0.594	NA	NA
Tested two or more times in last year (N = 692)						
Yes (n = 323)	30	9.3	4.10 (1.91–8.76)	<0.001	2.13 (0.93–4.88)	0.074
Number of casual^b^ condomless partners in last year (N = 506)						
0 (n = 206)	9	4.4	Reference	<0.001	Reference	0.007
1 (n = 154)	4	2.6	0.58 (0.18–1.93)		0.60 (0.18–2.07)	
≥ 2 (n = 146)	24	16.4	4.31 (1.94–9.57)		**2.86 **(**1.17**–**6.98**)	
Chemsex^c^ (N = 732)						
Yes (n = 153)	23	15.0	4.28 (2.33–7.86)	<0.001	**2.31 **(**1.09**–**4.91**)	0.030
Had an STI in last year (N = 731)						
Yes (n = 143)	21	14.7	3.88 (2.10–7.15)	<0.001	1.58 (0.72–3.45)	0.251
Exclusively serosorting (N = 409)						
Yes (n = 303)	21	6.9	1.50 (0.55–4.09)	0.424	NA	NA
Oral fluid specimen result (N = 537)						
Ab negative (n = 500)	29	5.8	Reference	0.569	NA	NA
Ab positive (n = 37)	3	8.1	1.43 (0.42–4.95)		NA	

Ab: antibody; AOR: adjusted odds ratio; CI: confidence interval; NA: not applicable; OR: odds ratio; PrEP: pre-exposure prophylaxis; STI: sexually transmitted infection.

^a^ Adjusted model contains a total of 480 observations.

^b^ Casual partner is defined as a partner with whom the respondent has had sex with only once.

^c^ Chemsex is defined as use of any of the following drugs before or during sex; mephadrone, ketamine, gammahydroxybutrate (GHB) or methamphetamine in the last 12 months.

## Discussion

This bio-behavioural study of MSM in London found high rates of behaviours associated with increased risk of HIV transmission. Most men (60.1%) reported condomless sex in the last 3 months; most (59.3%) had had condomless sex with at least one casual partner in the previous 12 months; one in five reported having an STI in the previous 12 months; and one in five men reported chemsex in the last year, higher than most estimates observed in other studies [16-19]. Despite this, the number of new diagnoses in MSM in London is falling and according to estimates from surveillance data, rates of undiagnosed infection have decreased from 18% in 2013 to 10% in 2016 [5,20]. Given the absence of change in sexual risk behaviours these decreases signal that biomedical prevention initiatives may be having a greater impact on the incidence of new infections. While the sample size for detecting undiagnosed infection is small in this study, we observed that 13.2% (95% CI: 2.4–23.9) of men living with HIV were unaware of their infection.

The population of MSM surveyed demonstrated high awareness of the importance of regular testing. Nearly all men (96.2%) reported ever having an HIV test and unprecedented rates of men reported having had a test in the previous 12 months (69.7%) in line with patterns reported elsewhere [7]. MSM reporting condomless sex with large numbers of causal partners (i.e. > 10) were more likely to report more frequent testing. However, men who were older were less likely to report frequent testing and men reporting PrEP or chemsex use were no more likely to report two or more tests in past year than men not reporting PrEP or chemsex use (although this should be interpreted with caution as we were not powered to detect this). This suggests that there are important subgroups of MSM for whom greater efforts to promote messages of regular testing should be reinforced, including those reporting the highest risk behaviours. The findings on frequency of testing are particularly relevant in the light of recent decreases in HIV diagnoses in MSM in London and in this study half (48.7%) of the men reported having had more than one HIV test in the last year. In addition, MSM having regular HIV tests are also more likely to test for and therefore be diagnosed with other STIs, reducing duration of infection and thus onward transmission of other STIs.

While 12.9% of men reported having four or more tests in the previous 12 months, in keeping with national guidance for HIV testing in MSM at higher risk of HIV, less than half (45.6%) of those reporting two or more casual condomless partners reported testing four or more times in the same time period. We did not ask about timing of risk behaviours and testing but these findings suggest that further progress is required to increase the rate of frequent testing for all men at higher risk of HIV acquisition. A lower than recommended frequency of testing in at risk MSM has also been reported from three other surveys in the UK between 2011 and 2013 [21].

Some men at higher risk of HIV infection are self-selecting PrEP as an option for HIV prevention. The rate of PrEP use reported here (6.2%) is within the range observed in two previous studies measuring PrEP use in MSM in England, which found 2.1% and 8% of participants had or were currently using PrEP respectively [22,23]. It is important to note that the numbers of men reporting PrEP use was small in this study, and with a larger sample size some associations found to be significant in the univariate model may have remained significant in the multivariate model. PrEP use in this study was associated with increased rates of casual condomless anal sex partners and use of drugs associated with chemsex, suggesting that those using PrEP are likely to be assessing their own need correctly. Those reporting STI diagnoses in the past year were significantly more likely in univariate analysis to use PrEP, but this association disappeared once other factors such as chemsex were controlled for in the multivariate model. Since we did not ask about the number of STI tests individuals had, we do not know from this study if the increased STIs’ rates are indicative of more frequent testing or because PrEP users are more likely to exhibit risk factors related to higher levels of STI transmission. The PROUD PrEP trial [11] found higher rates of STIs in the PrEP intervention arm, though they were high in both arms, and the difference was not significant once STI testing rates were controlled for. However, men enrolled in the Amsterdam PrEP implementation trial had a significantly higher hepatitis C virus (HCV) infection prevalence than those in the general population of HIV negative MSM attending SH services [24].

In our study, a small proportion of those reporting PrEP use in the last year had not had an HIV test in the same time period and PrEP use was not associated with frequent testing in either multivariate model. It is a concern that those using PrEP from other sources than SH clinics, such as online may not be accessing the clinical monitoring recommended for safe PrEP use and the early detection of other STIs such as HCV [25]. PrEP use in England is evolving rapidly and will have changed since this study was conducted, not least as a result of the PrEP implementation trial (IMPACT) which began recruiting in October 2017 [13]. While data on eligibility for PrEP use were not collected in this study, we found 59.2% of HIV-negative men reported recent condomless sex. While imprecise, this may be a broad indication of the proportion of men in this study population who could have benefitted from PrEP.

Study limitations include that convenience sampling was conducted in a range of pre-agreed social venues. The men using these venues are unlikely to be representative of all MSM in London and those participating in the survey may not be representative of all MSM in those venues. MSM in convenience samples tend to be younger, more likely to identify as gay and more likely to report more sexual risk behaviours than MSM in probability samples [26]. The cross-sectional study design used here makes it difficult to infer the temporal association between risk factors and outcomes. Response rates were not collected systematically and are not recorded here, collected rates ranged from 44% to 100%. There was a discrepancy between the likelihood of men providing an oral fluid sample based on their HIV status and four men self-reporting their status as HIV positive did not have anti-HIV detected by the oral fluid specimen assay likely due to reduced oral fluid test sensitivity in those on longstanding ART. Preparatory validation work found a sensitivity of 92.1% therefore a small number of false positives were expected, these rates are acceptable for epidemiological studies but not for diagnostic work.

In conclusion, this study suggests that uptake of combination HIV prevention appears to be higher than in previous reports among MSM in London. Higher rates of repeat HIV testing, some evidence of decreased prevalence of undiagnosed HIV infection and the use of PrEP by some MSM engaging in higher risk behaviours are all likely to be linked to fewer HIV diagnoses. The IMPACT trial will increase the number of MSM using PrEP which should reduce HIV transmission further. However, results from these analyses also demonstrate that there is a continued need to reinforce messages about HIV testing and behavioural risk reduction for all MSM. Condom use continues to play an important role in HIV and other STI prevention among MSM and should remain a key component of health promotion messages. Further work is required to investigate and overcome the barriers to testing and condom use that still exist for some men and to ensure that the potential successes of combination prevention are repeated in all communities and all areas of the country.

## Supplementary Data

149000 Supplement S1
