# Supplementary material for: Combination prevention and HIV: a cross-sectional community survey of gay and bisexual men in London, October to December 2016
Source: Euro Surveill. 2019 Jun 20;24(25):1800312. doi: 10.2807/1560-7917.ES.2019.24.25.1800312 (PMC6593902; doi:10.2807/1560-7917.ES.2019.24.25.1800312)
Supplement: Supplement S1 [file 1800312_LOGAN_Supplement.pdf]

This supplementary material is hosted by *Eurosurveillance* as supporting information alongside the article 'Combination prevention and HIV: a cross-sectional community survey of gay and bisexual men in London, October to December 2016' on behalf of the authors who remain responsible for the accuracy and appropriateness of the content. The same standards for ethics, copyright, attributions and permissions as for the article apply. *Eurosurveillance* is not responsible for the maintenance of any links or email addresses provided therein.

24 In the last year, have you used any of the following drugs just before or during sex?: (tick all that apply)

- ❑ Amphetamine (speed)
- ❑ Cocaine
- ❑ Ketamine (Vitamin K, K, Special K)
- ❑ GHB/GBL (Gina, Liquid Ecstasy, Liquid G)
- ❑ Mephedrone (Drone, MCAT, meow meow)
- ❑ Meth Amphetamine (Crystal meth, ice/glass, Tina)
- ❑ Poppers (Amyl or other nitrite inhalants)
- ❑ Others (please specify) ....
- ❑ Don't know
- ❑ None of the above

25 In the last year, did you inject any of these drugs?

❑ Yes ❑ No

26 Have you ever taken antiretroviral drugs after you had anal sex, to reduce the risk HIV infection? (also known as post-exposure prophylaxis or PEP)

❑ Yes ❑ No

If yes, how many times in the last year?

....

27 In the last 12 months, have you taken antiretroviral drugs before you had unprotected anal sex, to reduce the risk of HIV infection (also known as pre-exposure prophylaxis or PrEP)?

❑ Yes ❑ No

If yes, where did you get the medication from?

- ❑ A clinical trial e.g. PROUD
- ❑ Sexual health clinic
- ❑ A private prescription
- ❑ The internet
- ❑ Other

28 Are you currently taking PrEP?

❑ Yes ❑ No

29 In this study pack are a series of adverts and posters from the Do it London campaign. Have you seen any of these before?

❑ Yes ❑ No (Skip to 31)

If yes where (tick all that apply)?

- ❑ On London transport (e.g. tube or buses)
- ❑ Telephone boxes/billboards
- ❑ In newspapers/magazines
- ❑ On Facebook or twitter
- ❑ In App advertising e.g. Grindr
- ❑ From a Google search
- ❑ At Pride
- ❑ In a venue
- ❑ Other...

30 On seeing any of these specific messages/ adverts did you do any of the following? (tick all that apply)

- ❑ Visit the website: doitlondon.org
- ❑ Share the links/messages online
- ❑ Order condoms
- ❑ Use condoms during sex
- ❑ Discuss the campaign with friends or partners
- ❑ Visit a sexual health clinic
- ❑ Think about having a HIV test
- ❑ Discuss HIV testing
- ❑ Find out where I can get an HIV test
- ❑ Have an HIV test
- ❑ None of the above

31 In this study pack there is a picture of a Do It London condom and lube pack. Do any of the following apply to you? (tick all that apply)

- ❑ I have seen the Do It London condom and lube packs before
- ❑ I have taken Do It London condom and lube packs
- ❑ I have used Do It London condom and/or lube
- ❑ I have not seen any Do It London condom and lube packs

GAY MEN SEXUAL HEALTH SURVEY: 2016

QUESTIONNAIRE

Venue ______________________

Date ______________________

Conducted by:
University College London and Public Health England

Funded by:
National Institute for Health Research School for Public Health Research and London-wide HIV Prevention Programme

Please tick, number or write as appropriate

1 Have you already completed this questionnaire in the last three months?

- ❑ Yes ❑ No

If yes, where? (end here, thank you)

....

2 What was your age at your last birthday?

....

3 Which of the following ethnic groups best describes you?

- ❑ White
- ❑ Black (African/Caribbean/British/Other)
- ❑ South East Asian
- ❑ Asian (Indian/Pakistani/Bangladeshi)
- ❑ Latin American
- ❑ Mixed
- ❑ Other (please specify): ....

4 In which country were you born? (please specify)

....

5 Which borough/council do you live in? (e.g. Lambeth, Hackney, Islington)

....

6 Are you in paid employment at present?

- ❑ Yes ❑ No

7 How many years of full-time education have you had since you were 16?

- ❑ None ❑ Up to 2 years
- ❑ 2 years or more ❑ Still in full-time education

8 What do you believe to be your current HIV status?

- ❑ Negative ❑ Positive ❑ Don't Know

9 When did you have your last HIV test?

- ❑ In the last 3 months
- ❑ Between 3 and 12 months ago
- ❑ More than a year ago
- ❑ Over 5 years ago
- ❑ Never had an HIV test

10 In the last year, how many HIV tests have you had?

....

11 Where did you have your last HIV test?

- ❑ Sexual health/GUM clinic
- ❑ GP
- ❑ Self sampling service (took your own specimen and sent to laboratory)
- ❑ Self testing (tested yourself and got the result immediately)
- ❑ Other please specify ....

12 What was the result of your last HIV test?

- ❑ Negative (Skip to 14)
- ❑ Positive
- ❑ Don't know/result not collected (Skip to 14)

13 a) Are you currently on antiretroviral medication?

- ❑ Yes ❑ No

b) Your last HIV viral load was:

- ❑ Undetectable
- ❑ Detectable
- ❑ Don't know

14 In the last year, have you attended a Sexual Health/GUM clinic?

- ❑ Yes ❑ No

15 In the last year, have you had a STI?

- ❑ Yes ❑ No

If yes, which of the following STIs have you had (tick all that apply)

- ❑ LGV
- ❑ Gonorrhoea
- ❑ Shigella
- ❑ Syphilis
- ❑ Other (please specify) ....

16 How would you describe your sexual orientation?

- ❑ Gay/Homosexual
- ❑ Bisexual
- ❑ Straight/Heterosexual
- ❑ Other (please specify)....................................

17 a) In the last 12 months, how many men have you had sex with?

....

b) Of these how many were once only?

....

18 Have you ever had active/passive anal intercourse with a man?

- ❑ Yes ❑ No

19 When was the last time you had active/passive anal intercourse with a man without a condom?

- ❑ In the last 3 months
- ❑ Between 3 and 12 months ago
- ❑ More than 12 months ago

20 a) In the last year, with how many men have you had active/passive anal intercourse?

....

b) Of these how many were once only?

....

21 In the last year, with how many men have you had anal intercourse without a condom?

Of these:

a) how many were once only?

....

b) how many did you know had the same HIV status as you?

....

22 With these partners, were you:

- ❑ Always the active (top) partner
- ❑ Mostly the active (top) partner
- ❑ Equally both (versatile)
- ❑ Mostly the passive (bottom) partner
- ❑ Always the passive (bottom) partner

23 How do you usually meet your sexual partners (tick all that apply)

- ❑ In bars/clubs ❑ Through friends
- ❑ Gay dating apps /websites
- ❑ In Saunas or sex on-site premises
- ❑ Other
